# Factors associated with high-risk HPV infection and cervical cancer screening methods among rural Uyghur women aged > 30 years in Xinjiang

**DOI:** 10.1186/s12885-018-5083-1

**Published:** 2018-11-23

**Authors:** Sulaiya Husaiyin, Lili Han, Lin Wang, Chunhua Ma, Zumurelaiti Ainiwaer, Nuermanguli Rouzi, Mireguli Akemujiang, Hatiguli Simayil, Zumulaiti Aniwa, Rouzi Nurimanguli, Mayinuer Niyazi

**Affiliations:** Department of Gynecology, Xinjiang Uyghur Autonomous Region Peoples Hospital, No 91 Tianqi Road, Urumqi, Xinjiang, 830001 China

**Keywords:** Human papillomavirus infection, Colposcopy, Cervical epithelial lesion, Cervical cancer, Uyghur

## Abstract

**Background:**

Cervical cancer is the most common genital malignant tumor in women worldwide. However, the reliability of different detection methods may vary according to populations and epidemics. This study analyzed factors relevant to high-risk human papillomavirus (hrHPV) infection among rural Uyghur women aged > 30 years and evaluated the value of different screening methods for cervical precancerous lesions.

**Methods:**

From July 2015 to May 2016, 225 rural Uyghur women aged > 30 years were recruited from local health clinics throughout Pishan, Xinjiang, China. HrHPV DNA testing, colposcopy, biopsy of cervical precancerous lesions, and surveys were conducted. The results of different screening methods were compared, and factors associated with hrHPV infection were analyzed.

**Results:**

The rates of hrHPV infection and cervical epithelial lesions were 9.3 and 1.8%, respectively. The area under the ROC curve was 0.538 (95% CI: 0.292, 0.784; *P* = 0.753) for the HPV test and 0.995 (95% CI: 0.988, 1.003; *P* < 0.001) for colposcopy. Factors associated with HPV infection included widowhood (OR = 13.601 (2.170, 85.263), *P* = 0.005) and ≥ 3 sexual partners in the past 5 years (OR = 16.808 (4.148, 68.101), *P* < 0.001). .

**Conclusions:**

Among rural Uyghur women aged > 30 years, the main factors for HPV infection include marriage and frequent sexual intercourse. Colposcopy has a higher screening value for cervical epithelial lesions than hrHPV testing.

## Background

Cervical cancer is the most common malignant genital tract tumor in women and has shown an increasing incidence in recent years. The age of onset for cervical cancer tends to be young. Every year, approximately 500,000 new cases of cervical cancer occur worldwide, 90% of which are in developing countries [1]. China, the world’s largest developing country, exhibits a high incidence of cervical cancer; with approximately 130,000 new cases each year, the incidence in China accounts for 28.8% of the new cases worldwide. Furthermore, approximately 80,000 people die of cervical cancer in China each year; among tumor-induced deaths in Chinese females, this mortality ranks second only to mortality due to breast cancer [2, 3]. The Xinjiang region of China shows a high incidence of cervical cancer, and Uyghur women in particular exhibit a significantly high risk of cervical cancer. The prevalence of cervical cancer is significantly higher in Uyghur women than that in any other ethnic group; the incidence of cervical cancer among Uyghur women in Xinjiang has been reported to be 459/100,000–527/100,000, which is significantly higher than the incidence in any other region of China [4–6]. Multiple factors, such as human papillomavirus (HPV) infection, marriage and childbearing, contribute to the high incidence of cervical cancer among this population [7]. Compared with other female cancers recorded in this region, cervical cancer is the leading cause of death in Xinjiang women and is an important factor that influences the health and life expectancy of women.

The development of cervical cancer is continuous and progresses from cervical intraepithelial neoplasia (CIN) to early invasive cancer and then to more established cancer, which involves a long and reversible precancerous lesion stage. If CIN can be recognized early and then properly treated during this precancerous lesion stage, the conversion from CIN to early invasive cancer (consisting of quantitative changes leading to qualitative changes) and, thus, the development of cervical cancer can be prevented [8]. Standardized preventive cancer screenings and timely detection and treatment of cervical lesions can reduce the incidence and mortality of cervical cancer and are therefore essential to the prevention and treatment of cervical cancer, in addition to representing the most health-efficient and economical control strategies. Therefore, early cancer prevention screenings are very important. There are currently many approaches for early prevention screening for cervical cancer; the commonly employed methods include Pap smears, the ThinPrep cytology test (TCT), and colposcopy, but each approach presents its own advantages and disadvantages. Pap smears exhibit a high false-positive rate due to factors related to sampling and preparation [9]. Although TCT displays high sensitivity, it is also easily affected by subjective factors and the reader’s experience, while colposcopy is complex and can cause certain traumatic injuries [10]. In rural areas of many countries with limited medical and health resources, such as China, there is a clear need for highly specific and sensitive screening methods to improve the efficiency of cervical cancer screening and thereby ensure early detection, diagnosis, and treatment.

Some scholars have proposed the utilization of pathogeny screening, such as high-risk human papillomavirus (HPV) testing, as an early screening strategy [11, 12]. HPV infection is well known to be the main cause of cervical cancer, and the more severe the precancerous lesions, the higher the rate of HPV infection [13, 14]. More than 100 types of HPV have been identified, all of which are classified as high risk or low risk. More than 10 high-risk HPVs have been confirmed to be closely related to the development of cervical cancer and precancerous lesions and have become the main risk factors for cervical cancer and precancerous lesions; furthermore, almost all patients with cervical cancer or atypical hyperplasia are infected with HPV [15]. Although most infections may subside naturally, some persistent infections will progress to atypical hyperplasia, which leads to varying degrees of precancerous lesions and cervical cancer. Some studies have found a relatively high rate of HPV detection in cervical cancer tissues among Uyghur women, especially for HPV-16 [16, 17]. Hence, if the factors correlated with HPV infection can be determined, practical and effective preventive measures to reduce HPV infection in women could be developed, thereby reducing the incidence of cervical cancer. These types of measures constitute a preventive strategy that targets pathogenic causes of the disease. Risk factors for HPV infection have been reported based on studies from other regions [18, 19], but reports on Uyghur women are rare. Investigations of this population are undoubtedly meaningful for enriching the data related to cervical cancer and enhancing the understanding of this condition.

In this study, high-risk HPV DNA testing, colposcopy, cervical biopsy, and questionnaires were utilized to analyze the factors related to high-risk HPV infection and to evaluate the clinical value of applying different methods, using histopathology as the gold standard, to cervical precancerous lesion screening among normal rural Uyghur women in Xinjiang. The results may provide a reference for the prevention and treatment of cervical cancer in this population, as well as in populations in the regions with similar medical circumstances.

## Methods

### Subjects

In this study, a cross-sectional survey was conducted from July 2015 to May 2016 in Pishan County, Hotan Prefecture, in the Xinjiang Uyghur Autonomous Region of China. Information and motivation were provided by local health care workers in individuals’ homes to recruit local eligible women to participate in the survey. The inclusion criteria were as follows: women from rural areas, age older than 30 years, Uyghur ethnicity, and a history of sexual intercourse. The exclusion criteria were pregnancy or termination of pregnancy less than 3 months prior to the study; a past history of cervical cancer or unknown uterine tumors; a history of hysterectomy, cervical conization, radiotherapy or chemotherapy; cervical dysplasia; no sexual intercourse; mental illness or other serious disease; a long-term out-of-home residence; and women belonging to a nomadic population.

This study was approved by the Ethics Committee of the Xinjiang Uyghur Municipal People’s Hospital, and written informed consent was obtained from each participant.

### Methods

**Fig. 1:**
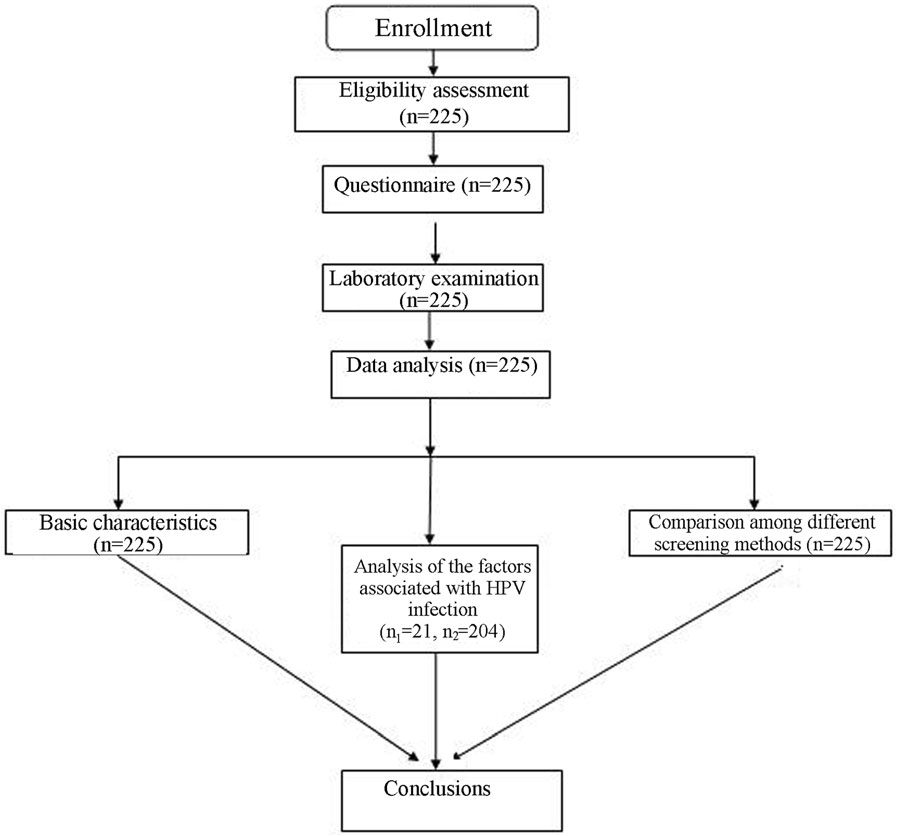
Flow chart of the experiments

All examinations of women participating in the study were conducted during nonmenstrual phases. These evaluations included a physical examination, a questionnaire, high-risk HPV DNA testing, colposcopy, and tissue biopsy. Respondents were not allowed to use vaginal medication or perform vaginal washing 3 days before the examination and could not engage in sexual behavior within 24 h. A detailed flow chart of the study design is shown in Fig. 1.

### Questionnaire

One-on-one interviews were conducted by trained investigators. The interview was performed according to a simple self-designed questionnaire that included basic demographic information, family background, marital status, childbearing status, gynecological conditions, and family history.

### High-risk HPV DNA screening method

A trained gynecological clinician used a speculum to widen the vagina of each subject and fully expose the cervix, cleaned away secretions using sterile saline cotton balls or dry cotton swabs and inserted a sampling brush (Digene Company, Gaithersburg, MD, USA) into the cervical orifice, rotating it clockwise 3–5 times. The sampling brush was slowly withdrawn and immersed in an HPV DNA sampling tube (Digene Company; a cell preservation solution was included in the tube), and the cap was tightened for storage. The sample was then sent for immediate detection; samples that could not be tested immediately were stored at 4 °C for testing within 2 weeks.

HPV DNA testing was conducted using the second-generation hybrid capture method to detect HPV-16, 18, 31, 33, 35, 39, 45, 51, 56, 58, 59, and 68, employing a DML 2000 gene recombination signal amplifying system and a DML 2000 microplate reader (Digene Company). The tubes were shaken well and numbered, and five drops of the indicator were applied to the lysate, followed by mixing. Approximately 1000 μl, 500 μl, and 500 μl of the lysate was applied to the negative control (2 ml), positive control (1 ml) and specimen, respectively, to maintain a consistent lysate concentration among the groups, and the samples were mixed. The solutions were subsequently placed in a water bath at 65 °C for 45 min, and all solutions appeared purple. The prepared probe B (25 μl) was added to a 96-well plate, and the lysate controls and samples (75 μl) were then added to the wells. Adhesive film was attached, and the samples were oscillated at 1100 rpm/min for 3 min. The solutions turned yellow and were then incubated at 65 °C for 60 min. Thereafter, the tape was removed, and the solution was cooled for 5 min. Approximately 100 μl of the solution was transferred to the capture wells, and tape was attached. Oscillation at 100 rpm/min was performed for 60 min. The liquid was then removed, and detection reagent 1 (75 μl) was added to the wells. The wells were subsequently capped, and the plate was placed at 20–25 °C for 30 min. The wells were then covered with absorbent paper, and the liquid was removed via rapid inversion. The plate was blotted on absorbent paper until dry. After washing six times, the sample was placed on absorbent paper, and blotting and drying were performed again. Approximately 75 μl of detection reagent 2 was subsequently added to each well, and the samples were incubated in the dark at 20–25 °C for 15 min. Finally, the results were read with a DML 2000 system. An HPV DNA concentration ≥ 1.0 pg/ml was considered HPV positive.

### Colposcopy and pathological biopsy

After vaginal secretion sampling (for HPV DNA testing), colposcopy (with colposcopes from Wallach, USA) was performed immediately by trained full-time practitioners with the subjects in a lithotomy position. The cervix was exposed with the aid of a disposable speculum. After secretions were cleared, the diopter and focal length of the colposcopic eyepiece were adjusted to display a clear image, and each area to be inspected was exposed. The appearance and color of the cervix were observed at low magnification, and the morphology of blood vessels was assessed using a green filter and high magnification. The cervix was compressed for 1 min with 3–5% glacial acetic acid solution (applying 1% compound iodine solution evenly to the cervix if necessary) to improve visualization of the cervical epithelium: the cervical squamous epithelium, columnar epithelium, and the color, appearance, and blood vessels of the transformation area were observed. Normally, abnormal or diseased regions are not colored or are mustard yellow in color. In cases categorized as CIN-I, the acetowhite epithelium was thin, with an irregular boundary and rare embedded and dotted blood vessels, and iodine coloring showed a negative result or resulted in yellowish coloring. For CIN-II, the acetowhite epithelium was thick and gray, with dotted and embedded blood vessels, and the coloring test showed a negative result or resulted in mustard yellow coloring. For CIN-III, the acetowhite epithelium was dense and gray, with irregular thick dotted blood vessels, and the coloring test showed a negative result or resulted in mustard yellow coloring. Live tissue samples from abnormal cervical regions were sent for pathological examination; if no suspicious lesions were found, tissues from the 3, 6, 9, and 12 o’clock sites of the transformation area were routinely obtained for biopsy, or endocervical curettage was performed. All living tissue samples were fixed with formaldehyde and labeled with the subject’s information before being sent for pathological examination.

Pathological diagnoses included the following: (1) normal cervical epithelium (including benign cervical epithelial cell changes); (2) CIN-I (only mild dysplasia or abnormal cell growth in the cervical epithelium); (3) CIN-II (moderate dysplasia confined to the basal two-thirds of the epithelium); (4) CIN-III (severe dysplasia spanning more than two-thirds of the epithelium, potentially involving the full thickness – such lesions may sometimes also be referred to as cervical carcinoma in situ); and (5) cervical squamous cell carcinoma (SCC) [20].

### Statistical analysis

A database was established using EpiData through double data entry and verification. The data were processed and statistically analyzed using SPSS 19.0. The histopathological findings were used as the gold standard, and the sensitivity, specificity, accuracy, positive likelihood ratio, and Youden’s index of the different detection methods were calculated. The effects of the different screening methods for cervical precancerous lesions were compared, and receiver operating characteristic (ROC) curves were drawn. The factors associated with HPV infection were analyzed through univariate and multivariate stepwise logistic regression analyses. *P* < 0.05 was considered to indicate statistical significance.

## Results

### Basic information

A total of 225 eligible women were enrolled in the study, with an average age of 43.6 ± 8.1 years; the minimum age was 31, and the maximum age was 74 years. The characteristics of these enrolled women are summarized in Table 1. Overall, there were 21 cases of high-risk HPV, and the infection rate was 9.3%.

**Table 1:** Characteristics of the 225 rural Uyghur women included in the study

Items	Percentage (%)	Items	Percentage (%)
Age		Age at first marriage	
*31–40 years old*	79 (35.1)	*≤16 years old*	65 (28.9)
*41–50 years old*	110 (48.9)	*17–18 years old*	113 (50.2)
*> 50 years old*	36 (16.0)	*> 18 years old*	47 (20.9)
Education		Number of times married	
*Primary school or lower*	153 (68.0)	*Once*	129(57.3)
*Junior high school*	66 (29.3)	*≥Twice*	74 (32.9)
*Senior high school and higher*	6 (2.7)	*≥Three*	22 (9.8)
Annual household income		Number of pregnancies	
*≤1000 Yuan*	71 (31.6)	*≤2*	40 (17.8)
*1001–2000 Yuan*	76 (33.8)	*3–4 times*	87 (38.7)
*> 2000 Yuan*	78 (34.7)	*> 4 times*	98 (43.6)
Marital status		Number of sexual partners in the past 5 years	
*Married*	212 (94.2)	*1*	199 (88.4)
*Separated*	2(0.9)	*2*	15 (6.7)
*Divorced*	5 (2.2)	*≥3*	11 (4.9)
*Widowed*	6 (2.7)	Age of menarche	
Menopause (%)	71/225 (31.6)	*≤13 years old*	18 (8.0)
Family history of cervical cancer	10/225 (4.4)	*13–16 years old*	167 (74.2)
		*> 16 years old*	40 (17.8)

The colposcopic results showed that there were 217 normal cases, 6 cases of CIN-I, 1 case of CIN-II, and 1 case of CIN-III in our study population. The biopsy results identified 221 normal cases, 3 cases of CIN-II, and 1 case of CIN-III, and the corresponding prevalence rate of high-grade CIN (II and III) was 1.8%.

### Comparison between the results of HPV infection tests and histopathological results

CIN II and III were recorded as positive pathological results (+), while other pathological findings were recorded as negative (−). The pathological results were used as the gold standard, and the comparison between the HPV results and the gold standard is shown in Table 2. The sensitivity of HPV testing was 25.00%; specificity, 90.95%; accuracy, 89.78%; positive predictive value, 4.76%; negative predictive value, 98.53%; Youden’s index, 0.16; positive likelihood ratio, 2.76; and negative likelihood ratio, 0.82.

**Table 2:** Comparison of results among HPV testing, colposcopy and histopathology

Screening test	Results	CIN		Total
		+	–	
HPV	+	1	20	21
	–	3	201	204
Colposcopy	+	4	4	8
	–	0	217	217
Total		4	221	225

### Comparison between colposcopy results and histopathological results

**Fig. 2:**
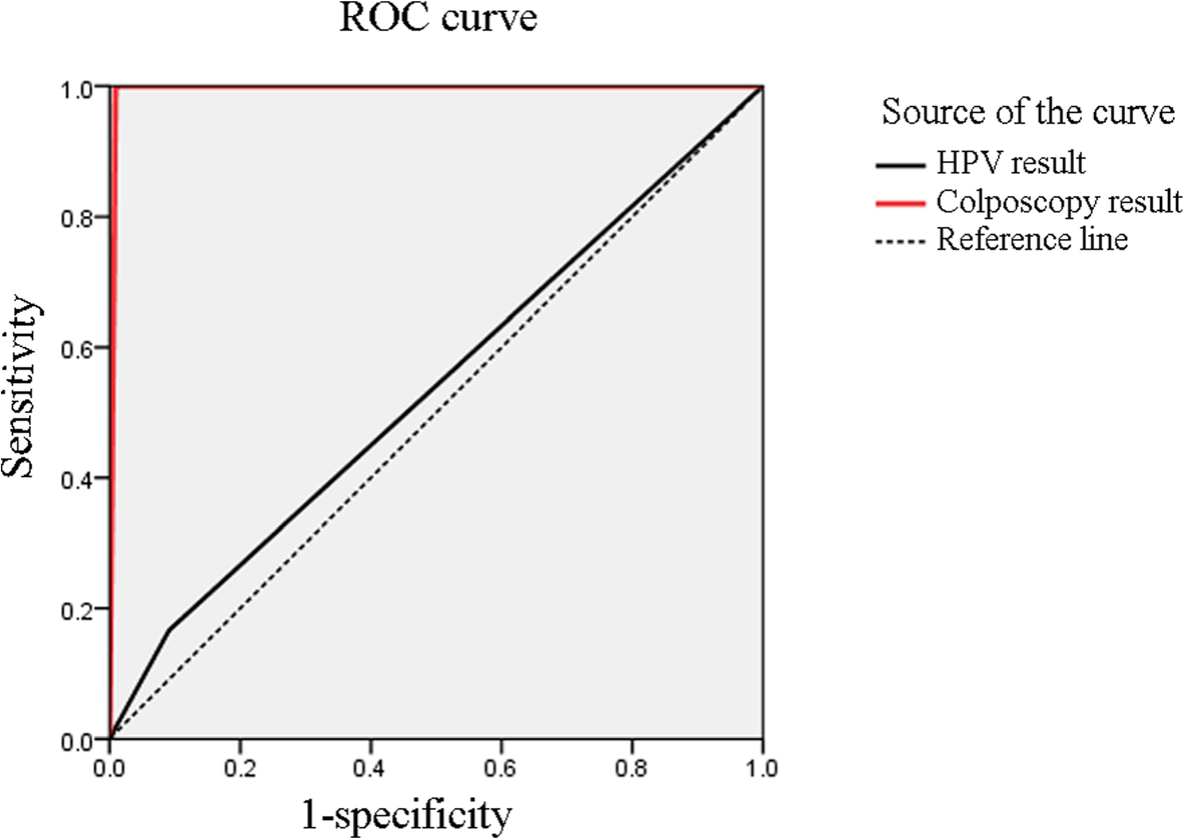
ROC analysis of the HPV test and colposcopy results

Similarly, for analyses of the colposcopy results, CIN II and III were recorded as positive pathological results (+), while other pathological findings were recorded as negative (−), and the pathological results were used as the gold standard. The comparison between the colposcopy results and the gold standard is shown in Table 2. The sensitivity was 100%; specificity, 98.19%; accuracy, 98.22%; positive predictive value, 50%; negative predictive value, 100%; Youden’s index, 0.98; positive likelihood ratio, 55.25; and negative likelihood ratio, 0.76. Using the biopsy results as the gold standard, the area under the ROC curve for the HPV test was 0.538 (95% CI: 0.292, 0.784), *P* = 0.753, while the area under the ROC curve for colposcopy was 0.995 (95% CI: 0.988, 1.003), *P* < 0.001. Further details are shown in Fig. 2 and Table 3.

**Table 3:** ROC analysis of HPV test and colposcopy results (biopsy results were used as the gold standard)

Tests	Area under the curve	*P* value
HPV test	0.538 (0.292, 0.784)	0.753
Colposcopy	0.995 (0.988, 1.003)	< 0.001

### Comparison between the results of HPV infection tests and colposcopy

A paired χ^2^ test was performed to compare the HPV results and colposcopy results, and a significant difference was observed between them (χ^2^ value = 5.333, *P* = 0.021; Table 4).

**Table 4:** Comparison of results between the HPV test and colposcopy

HPV test results	Colposcopy		χ^2^ value	P value
	Negative	CIN		
Negative	197	7	5.333	0.021
Positive	20	1		

Based on paired χ^2^ test

### Analysis of the factors associated with HPV infection

The HPV test and pathological examination results were included as dependent variables, and the other factors were used as independent variables. Univariate logistic regression analysis and multivariate stepwise logistic regression analysis were then performed successively. The rejection standard for including an independent variable in the stepwise logistic regression analysis was 0.1.

Among the examined factors associated with HPV infection, those that showed significant differences in the univariate analysis were marital status, number of times married, number of sexual partners in the past 5 years. The following factors showed significant differences in the multivariate analysis: being widowed (OR = 13.601 (2.170, 85.263), *P* = 0.005), having ≥3 sexual partners in the past 5 years (OR = 16.808 (4.148, 68.101), *P* < 0.001) (Table 5).

**Table 5:** Analysis of factors relevant to HPV infection

Items	Multivariate analysis of HPV infection	
	OR (95.0% CI)	P
Marital status		
*Married*	1	
*Separated*	13.092 (0.676, 253.422)	0.089
*Divorced*	7.545 (0.904, 63.006)	0.062
*Widowed*	13.601 (2.170, 85.263)	0.005
Number of sexual partners in the past 5 years		
*1*	1	
*2*	2.137 (0.365, 12.503)	0.400
*≥3*	16.808 (4.148, 68.101)	< 0.001

## Discussion

One of the measures currently used to improve the prevention and management of cervical cancer in women is enhancement of etiological analysis and prevention, where various factors are analyzed to determine the factors that are primarily associated with the disease; these factors are then targeted in prevention efforts. Another approach is to perform early, standardized screening in women in high-risk areas to identify and eliminate the factors leading to cervical cancer, such as precancerous lesions, with the goal of reducing the incidence of cervical cancer and prolonging patient survival through early detection, diagnosis, and treatment.

There were three main findings of this study regarding the screening for cervical precancerous lesions in rural Uyghur women through HPV testing and colposcopy. First, the rate of high-risk HPV infection in rural Uyghur women aged > 30 years in Pishan County was 9.3%, and the high-grade CIN (II and III) rate was 1.8%. Second, the colposcopy results were significantly consistent with those of cervical biopsy in the diagnosis of CIN; additionally, colposcopy exhibited a high sensitivity and specificity, showing an area under the ROC curve of 0.995, indicating the diagnostic value of this approach. High-risk HPV DNA testing exhibited poorer performance in detecting CIN; although it showed high specificity (90.95%), the sensitivity was low (25.00%), and the area under the ROC curve was only 0.538, with no significant difference. Therefore, in the absence of pathological biopsies, colposcopy is a better approach for determining whether there are precancerous lesions in Uyghur women. Finally, the results of the stepwise logistic regression analysis showed that the relevant factors for high-risk HPV infection in rural Uyghur women were their widowed status, and their number of recent sexual partners (specifically, having more than one sexual partner in the past 5 years).

There are a variety of approaches that are currently used for early cervical cancer screening. HPV detection is an effective method of cervical cancer screening, showing a high sensitivity in screening for CIN-II and CIN-III, and may become the primary tool for cervical cancer screening in women over 30 years of age [21]. However, others believe that HPV testing cannot completely replace TCT because only persistent infections with carcinogenic HPV subtypes lead to cervical cancer, and the risk of cervical cancer induced by HPV infection is low, as most infections can be cleared automatically [22, 23]. For HPV-positive women, especially those younger than 35 years, if no abnormal findings are found through traditional examination methods, they can be prescribed only observation without further examination. In addition, the importance of the persistence of HPV infection should be emphasized in health promotion and education activities as well as in the development of strategies for cervical cancer diagnosis and treatment. Others have proposed that the combined application of HPV tests and TCT is the best screening method for cervical cancer and precancerous lesions [24]. The detection rate of cervical lesions via HPV testing was found to be only 30%, but the detection rate of cervical precancerous lesions increased to 90% when HPV tests were combined with TCT [25]. Another study indicated that the sensitivity of HPV tests combined with TCT was 97.5%; the negative predictive value reached 98.2%; and the sensitivity in detecting high-degree cervical lesions and cervical cancer was as high as 100% [26]. Liu et al. found that TCT combined with HPV testing showed a desirable sensitivity and specificity that could meet the requirements for early cervical cancer and precancerous lesion screening [27]. In this study, both the sensitivity and specificity of the HPV test for screening of precancerous lesions in Uyghur women were low.

Colposcopy is a widely used screening method for cervical disorders and plays an important role in the determination of disease. This methodology has been rapidly developed in recent years and, combined with the use of acetic acid or iodine and optical amplification technology, provides a clearer picture of the abnormal areas of cervical lesions. Biopsy under microscope is the “gold standard” for the diagnosis of CIN and cervical cancer and is an indispensable second-line screening tool in cervical cancer screening. Luo et al. reported that the sensitivity of colposcopy in diagnosing CIN was 84.2%, while the specificity was 51.6%, indicating a high predictive value [28]. In the present study, we found that the consistency between the colposcopy and pathological biopsy results was significant, suggesting that colposcopy can be used as a screening tool for precancerous lesions. However, colposcopy is also considered by some to present certain limitations that are difficult to overcome in clinical application, such as poor exposure of lesions within the cervical canal, which could easily lead to misdiagnosis. Other limitations of this approach include that it reveals only the local epithelial and vascular morphological changes caused by the lesions and is unable to depict the fine structure of the cell; that it only provides possible lesion sites, without determining the nature of the lesion; and that colposcopy and biopsy represent a traumatic type of examination that is not suitable for application in large-scale screening.

At present, the international community follows a three-step screening method: cervical cytology or cytology combined with HPV detection is performed as the preliminary screening; cases that are suspicious or positive are referred to colposcopy; and colposcopic biopsy and pathological examination of the lesions are then performed. The China Cancer Research Foundation has proposed three programs: the preferred plan (TCT combined with HPV testing), the common plan (cervical vaginal cytology smear and HPV detection), and the basic plan (visual inspection of the lesion combined with the acetic acid or iodine test). When selecting the screening method, the optimal allocation of medical resources in different regions and the risk of disease development in different populations should be considered; costs, techniques, screening sites and other objective conditions should also be considered. Furthermore, good screening methods must include a rational scientific strategy for patients showing negative results in preliminary screening, to provide guidance regarding their need for follow-up and decide upon the review intervals to capitalize on the role of screening, in addition to reducing misdiagnosis and missed diagnoses and minimizing the threat of cervical cancer to the lives and wellbeing of women.

In this study, biopsy revealed a detection rate of high-grade CIN (II and III) of 1.8%. In a previous investigation of 20,000 women in Shanxi, China, 410 (2.05%) of the women were diagnosed histologically with CIN lesions, including 317 (1.58%) diagnosed with CIN-I, 93 (0.50%) with CINII/III, and 11 (55/100,000) with squamous cell carcinoma (SCC) [29]. In another study conducted on 316 women aged 18–25 years in Jiangsu, China, 3.4% of the study subjects were diagnosed with CIN-II [30]. Our results are basically consistent with those reported previously.

The development of cervical cancer is associated with various factors, including viral infection, sexual behavior, and education, and studies have shown that high-risk HPV infection is a major risk factor for cervical cancer [20, 31]. More than 90% of cervical cancers are associated with high-risk HPV infection. Persistent HPV infection is a necessary cause of cervical cancer and precancerous lesions. Additionally, 10–15% of adult women over 35 years of age with continuous HPV infection have been found to exhibit a higher risk of developing cervical cancer; however, the occurrence of cervical cancer also depends on the virus, host, and environment, along with their mutual synergies [32]. Sexual behaviors have been widely investigated in this context, including an increased number of sexual partners and premature sex, which increase an individual’s predisposition to HPV infection; contraceptive administration affects HPV susceptibility as well [33–35]. In this study, marital status and the number of sexual partners were associated with HPV infection. Uyghur women tend to engage in relatively conservative sexual practices, and their sexual partners were therefore mainly their husbands, with extra-marital sexual behavior being relatively rare. Thus, the marital status of these women could indirectly reflect their sexual behavior, such as their number of sexual partners; widowed women were also prone to have more sexual partners. This conclusion is consistent with those of previous studies [33–35]. This study also indicated that marital status, which reflects sexual behavior, was a factor associated with HPV infection, suggesting that we should consider whether sexual behavior is a factor that is indirectly relevant to precancerous lesions.

## Conclusions

In conclusion, colposcopy was found to be more effective than high-risk HPV testing in screening for cervical precancerous lesions among rural Uyghur women in Pishan County, and this method can be popularized for cervical precancerous lesion screening in future preventative and curative practices. Factors related to sexual life, including marital status, were the main factors correlated with HPV infection among these women. However, as the conclusions of this study were mainly obtained through the investigation of Uyghur women in Pishan County, extrapolation of the findings should consider the representation of the samples and the differences between different regions. Furthermore, the factors associated with cervical precancerous lesions were not analyzed due to a small case number of high-grade CIN. To overcome this limitation, multi-center studies with a larger sample size are still needed.

## Abbreviations

CIN: Cervical intraepithelial neoplasia
HPV: Human papillomavirus
OR: Odds ratio
ROC: Receiver operating characteristic
SCC: Squamous cell carcinoma
TCT: ThinPrep cytology test
